# An approach to identifying drug resistance associated mutations in bacterial strains

**DOI:** 10.1186/1471-2164-13-S7-S23

**Published:** 2012-12-07

**Authors:** Michal Wozniak, Jerzy Tiuryn, Limsoon Wong

**Affiliations:** 1 Faculty of Mathematics, Informatics and Mechanics, University of Warsaw, Poland; 2 School of Computing, National University of Singapore, Singapore

## Abstract

**Background:**

Drug resistance in bacterial pathogens is an increasing problem, which stimulates research. However, our understanding of drug resistance mechanisms remains incomplete. Fortunately, the fast-growing number of fully sequenced bacterial strains now enables us to develop new methods to identify mutations associated with drug resistance.

**Results:**

We present a new comparative approach to identify genes and mutations that are likely to be associated with drug resistance mechanisms. In order to test the approach, we collected genotype and phenotype data of 100 fully sequenced strains of *S. aureus *and 10 commonly used drugs. Then, applying the method, we re-discovered the most common genetic determinants of drug resistance and identified some novel putative associations.

**Conclusions:**

Firstly, the collected data may help other researchers to develop and verify similar techniques. Secondly, the proposed method is successful in identifying drug resistance determinants. Thirdly, the *in-silico *identified genetic mutations, which are putatively involved in drug resistance mechanisms, may increase our understanding of the drug resistance mechanisms.

## Introduction

The problem of bacterial drug resistance did not exist in 1930s, when antibiotics were introduced to treat bacterial infections. Since then, due to various factors--such as irresponsible dosage of antibiotics, naturally occurring mutations, transmission of drug-resistant strains, etc.--drug resistance has become a serious health problem. This has drawn the attention of WHO (World Health Organization), ECDC (European Centre for Disease Prevention and Control) and CDC (Centers for Disease Control and Prevention), which monitor and report the spreading of drug-resistant pathogens in the world. As a consequence, for example, WHO launched in 2006 a new global program "Stop TB Strategy" to fight the spreading of *M. tuberculosis *(MTB). A recent WHO report on MTB estimates that the bacteria was responsible for around 1.7 million deaths world-wide in 2009 [1]. According to the report, 3.3% of new MTB cases in 2009 were multi-drug resistant (MDR). Moreover, 58 countries reported cases of extensively-drug-resistant (XDR) isolates of the bacteria. Very recently, ECDC reported that as high as 58% of all *Staphylococcus aureus *isolates tested in Malta was methicillin resistant (MRSA) [2].

The emergence of drug resistance is appalling, because it is often not economically justifiable for pharmaceutical companies to develop new drugs against it [3]. One promising approach to address the problem is to use old drugs that were designed for treating other diseases and are also effective against pathogens [4]. An effort in this direction was recently undertaken in a research study on *M. tuberculosis *[5]. The authors used three-dimensional docking to identify *in-silico *some putative drug-target interactions. For example, they predicted Comtan, a drug used in treating Parkinson's disease, as potentially effective against *M. tuberculosis *infections.

The need of more efficient strategies to develop new drugs stimulates research to better understand drug resistance mechanisms. Several drug resistance mechanisms have been discovered so far. They can be categorized as: (i) drug target modification; (ii) drug molecule modification by specialized enzymes; (iii) reduced accumulation of the drug inside a bacteria cell by decreased cell wall permeability or by pumping out the drug; and (iv) alternative metabolic pathways [6]. Moreover, there are known genes and mutations responsible for most of the drug resistance mechanisms. While genomics can be used on samples before and after drug resistance emerges to identify the likely associated mutations, most of the known mutations and genes associated with drug resistance were discovered by analyzing *a priori *candidates such as drug target genes or genes located on plasmids. Some information on drug target genes is available in the drugbank.ca database [7]; and some lists of genes known to be responsible for drug resistance (specific to bacterial species and drugs) are available in the ARDB (Antibiotic Resistance Genes Database) database [8].

Despite the above mentioned achievements, our understanding of drug resistance mechanisms is still incomplete. For example, there are reports of *S. aureus *isolates with atypical drug resistance profiles [9-11], which have not been explained yet. We hypothesize that these atypical drug resistance profiles might be due to genomic mutations in genes which are not *a priori *suspected of being involved in drug resistance mechanisms.

In this work, we use whole-genome sequences to identify and associate genetic mutations with drug resistance phenotype for bacterial strains (within *S. aureus*). Thus, conceptually our approach is similar to Genome-Wide Association Study (GWAS) approaches, which have been successfully applied to identify SNPs associated with human diseases [12,13]. We hypothesize that similar approaches, when applied to bacteria, should bring interesting results. However, it may not make sense to directly transfer this methodology to bacteria, because, for example, horizontal gene transfer (HGT) plays an important role in the evolution of bacteria. Besides mutations which would explain the reported atypical drug resistance profiles, we expect, by applying our approach, to identify also mutations that can be interpreted as compensatory mutations. These compensatory mutations are not directly involved in drug resistance, but they are important to neutralizing the deleterious effect (caused by mutations directly responsible for the resistance mechanisms) on bacterial fitness [14-16].

There are published studies, based on comparative analysis of whole-genome sequences, associating genetic mutations with drug resistance [17-21]. However, the methodologies used in these studies are simple and were applied to a relatively small number of strains. In our opinion, this is caused by two main problems: first, the number of fully sequenced bacterial strains within the same species have not been sufficiently large until recently; second, phenotype data with respect to drug susceptibility tests are spread throughout the literature and are not easy to collect.

In this work, we collected genotype data for 100 fully sequenced *S. aureus *strains and addressed the second problem by a careful search of the literature for results of drug susceptibility tests of the strains considered. We also developed and tested a new approach to associate mutations and genes with drug resistance.

## Materials and methods

Below we present details of our methodology including the problem setting, collection of data and subsequent steps of the identification of drug resistance associated genetic features. These subsequent steps comprise:

• unification of protein-coding gene annotations of bacterial strains and determination of gene families;

• computing multiple alignments for the gene families and reconstructing the consensus phylogenetic tree;

• identification of genetic features, possibly associated with drug resistance, such as point mutations and gene gain/losses, based on the multiple alignments and the determined gene families; and

• association of genetic features with drug resistance phenotypes.

### Problem setting

We consider a set S  of bacterial strains and their response to the application of a given drug. The response, which we called *drug resistance profile*, is represented by a vector $v:S\to {\{}^{\prime }{\text{S}}^{\prime }{,}^{\prime }{\text{R}}^{\prime }{,}^{\prime }{?}^{\prime }\}$, where by 'S' and 'R' we denote respectively drug-susceptible and drug-resistant strain phenotypes, by '?' we indicate that the phenotype is unknown. Additionally, we denote by $S_{v}^{S}$ and $S_{v}^{R}$ the sets of drug-susceptible and drug-resistant strains for a given drug resistance profile, respectively.

We also assume to have a given set of genetic mutations among the considered bacterial strains. Analogous to drug resistance profiles, we represent mutations as vectors $m:S\to \sum \cup \left\{{}^{\prime },{?}^{\prime }\right\}$, where ∑ denotes an alphabet of possible states, such as amino acids in the strain sequence corresponding to a given position in the multiple alignment. By '?' in the mutation profile we denote strains which are not present in the aligned gene family corresponding to the considered point mutation.

Then, the problem is mainly to identify a subset of genetic mutations associated with a given drug resistance profile and, secondarily, to use the identified mutations to predict unknown places in the given drug resistance profile (marked by '?').

### Genotype data

We collected genotype data (genome sequences and annotations) for the following 100 fully sequenced strains of *S. aureus *from the GenBank [22] and PATRIC databases [23]. Additionally, genotype data for strain EMRSA-15 were downloaded from the Wellcome Trust Sanger Institute website. At the time of writing, 31 out of the 100 *S. aureus *strains had "completed" sequencing status. For the remaining strains whose genomes are still being assembled, contig sequences (covering around 90% of the genomes) and annotations are provided.

We unify the original annotations employing our previously published method, called CAMBer [24]. Briefly, CAMBer iteratively extends the protein-coding annotations by homology transfer, until the transitive closure of a given homology relation is computed. This homology relation defines the consolidation graph. In this graph, there is an edge between a pair of genes if there was an accepteble BLAST hit between them.

Then, we determine gene families as connected components in the consolidation graph. However, we additionally extend the consolidation graph by edges coming from BLAST amino-acid queries. More formally, we add an edge between a pair of genes to the consolidation graph if the percent of identity (calculated as the number of identities over the length of the longer gene) of the BLAST hit between them exceeds a threshold *P*(*L*) given by the HSSP curve formula [25]:

(1) $$P\left(L\right)=\left\{\begin{array}{cc} 100 & L\leq 11\\ c+480.L^{\;-0.32.\left(1+e^{-L/1000}\right)} & 11<L\leq 450\\ c+19.5 & L>450 \end{array}\right)$$

Here, *c *is set to 40.5 and *L *is the number of aligned amino acid residues.

Each connected component in the consolidation graph corresponds to a gene family [24]. We compute multiple alignments using MUSCLE [26] for all these gene families. Then, we consider two kinds of genetic variations:

• gene gain/loss,

• amino acid point mutations.

Intuitively, we represent the considered genetic variations as 0 - 1 vectors, indexed by strains, where 0 denotes the reference state and 1 denotes some change. We call vectors of these genetic variations as gain/loss profiles and point mutation profiles.

Gene gain/loss profiles are transformed from gene families which do not span the set S  of all considered strains. For each such gene family, we transform it into a vector representation $g:S\to \left\{{}^{\prime },{\text{G}}^{\prime },{,}^{\prime },{\text{L}}^{\prime }\right\}$ as follows: for a given strain *i*, we define *g*(*i*) = 'G' if the gene family contains at least one gene in that gene family for strain *i*; otherwise we set *g*(*i*) = 'L'.

Similarly, point mutation profiles are transformed from columns in multiple alignments computed for gene families with elements present in at least |S|-1 strains. We take into account only columns which contain at least two different characters (ignoring '?'). For each such column (in the multiple alignment), we transform it into a vector representation $m:S\to \sum_{AA}\cup \;\left\{{}^{\prime },{-}^{\prime },{,}^{\prime },{?}^{\prime }\right\}$ as follows: for a given strain *i*, we set *m*(*i*) = 'x' if the character 'x' is present (one of 20 amino-acids or '-') in the row corresponding to strain *i*; and set *m*(*i*) = '?' if strain *i *is not present in the aligned gene family.

### Phylogenetic tree of the strains

We compute the phylogenetic tree of the input strains using a consensus method with majority rule implemented in the PHYLIP package [27]. We apply the consensus method to trees constructed for all gene families with exactly one element in each strain. The trees are constructed using the maximum likelihood approach implemented in the PHYLIP package [27].

### Phenotype data (drug susceptibility)

Drug susceptibility data were collected from the following sources: (i) publications issued together with the fully sequenced genomes: USA300_TCH1516 and USA300_TCH959 [28], MRSA252 and MSSA476 [29], 04-02981 [30], T0131 [31], ST398 [32], COL [33], JKD6008 [34], 16 K [35], TW20 [36], Newman [37], RN4220 [38], O46 and O11 [39], RF122 [40], Mu3 [41], MRSA252 [29], CF-Marseille [42], N315 and Mu50 [43], MW2 [44], MSHR1132 [45], ECT-R_2 [46], JH1 and JH9 [17], ED98 [47], JKD6159 [48], LGA251 [49], ED133 [50]; (ii) NARSA project http://www.narsa.net; (iii) email exchange with the authors of publications related to strains ST398 and TW20; and (iv) other publications found by searching of related literature [21,23,33,37,51-98]. The complete collected phenotype data are available in the supplementary table (additional file 1).

We represent the collected information as a set of drug resistance profiles, defined for each drug separately.

### Essential mutations

For a given drug resistance vector *v *we introduce a function *r_v _*which describes the reference state of a given point mutation or gene gain/loss profile *p*. We define it as the most often-occurring state in drug-susceptible strains (ignoring '?'), i.e.:

(2) $$r_{v}\left(p\right)=\underset{x\in \Sigma }{\text{arg}\text{max}}\underset{i\in S_{v}^{S}}{\sum }\left[p\left(i\right)=x\right]$$

Here, and in all the following equations, square brackets are used for Iverson's notation.

In the current implementation, in the case when there is a multiple number of states present in the same maximal number of strains, the function *r_v _*returns the first state in the lexicographical order. Note that this is just a technical assumption, since such mutations will not be considered as associated with drug resistance.

We say that a point mutation *m *is *present *in a strain *i *if *m*(*i*) ∉ {*r_v_*(*m*),'?'}; otherwise we say that the point mutation *m *is *absent *in strain *i*.

Then, we distinguish two categories of gene gain/loss and point mutation profiles depending on how they correspond to a given drug resistance profile. We categorize a given mutation profile *m *as:

• *essential mutation*, when *m *is absent in all drug-susceptible strains,

• *conflict mutation*, when *m *is present in at least one drug-susceptible strain.

Further, we distinguish *neutral mutations *as a subclass of *essential mutations*, these are *essential mutations *that are not present in any of drug-resistant strains.

Analogously, we transfer the above introduced concepts to gene/loss profiles, defining *essential, neutral *and *conflict *gain/loss profiles.

### Support

We aim to identify genetic variations which are likely to be associated with drug resistance. Intuitively, such mutations or gained genes should often be present in drug-resistant strains and rarely in drug-susceptible strains. To reflect this intuition we assign a score, which we call a *support*, to all point mutation and gene gain/loss profiles. For a given point mutation or gene gain/loss profile *p *and drug resistance profile *v*, the *support *(*s_v_*) is defined as the number of drug-resistant strains with the mutation present (or gene gained) minus the number of drug-susceptible strains with the mutation present (or gene gained):

(3) $$s_{v}(p)=\sum_{i\in S_{v}^{R}}\left[p(i)\neq r_{v}(p)\right]-\alpha_{v}\sum_{i\in S_{v}^{S}}\left[p(i)\neq r_{v}(p)\right]$$

Here, *α_v _*is a weight which we use to punish mutations for their presence in drug-susceptible strains. It is defined as the proportion of the number of drug-resistant to the number of drug-susceptible strains, so that occurrences of a mutation are given equal emphasis in drug-resistant and drug-susceptible strains. More formally:

(4) $$\alpha_{v}=\frac{\left|S_{v}^{R}\right|}{\left|S_{v}^{S}\right|}$$

### Weighted support

Although the *support *is a simple and intuitive score, it does not incorporate any phylogenetic information. For example, let us assume there are two point mutations with the same *support *3, where the first mutation covers only drug-resistant strains within one subtree of the phylogenetic tree, whereas the second mutation covers the same number of strains but spread throughout the whole tree. The first mutation is likely to be associated with the phylogeny, driven by some environmental changes. This suggests that the second mutation should have a greater score as it has to be acquired a few times independently during the evolution process.

We propose *weighted support *as a score to account for the above situation. For a given phylogenetic tree *T *and gene gain/loss or point mutation profile *p, weighted support *(*ws_v_*) is defined as follows:

(5) $$ws_{v}^{T}(p)=\sum_{i\in S}w_{i}^{T}[p(i)\neq r_{v}(p)]$$

where $w_{i}^{T}$ are weights assigned to each cell in a given drug resistance profile.

In all our experiments we assign weights in the following way: all drug-susceptible strains are assigned weight -*α_v _*(defined as above); each drug-resistant strain *i *is assigned a weight $\frac{1}{n}$, where *n *is the number of drug-resistant strains in the subtree (containing strain *i*) determined by its highest parental node, such that the subtree does not contain any drug-susceptible strain in its leaves. All strains without drug resistance information are assigned weights 0.

Note that the *support *score can also be expressed as *weighted support*, where *w_i _*are assigned as -*α_v_*, 1, 0 for drug-susceptible, drug-resistant and strains without drug resistance information, respectively.

**Figure 1:**
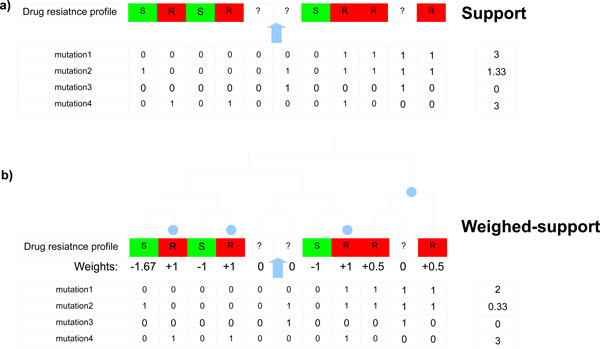
**Support and weighted support**. A schematic example of classification of genetic variation profiles and computation of their supports. Point mutations 1 and 4 are *essential*, mutation 2 is *conflict *and mutation 3 is *neutral*. Light blue circles mark nodes which appear in the definition of *weighted support*. These are nodes the highest parental nodes (for the leaf nodes corresponding to drug-resistant strains), that their subtrees do not contain any drug-susceptible strains in leaves. The scores (a) *support *and (b) *weighted support *are assigned to these mutations. For this drug-resistance profile, the ratio *α_v _*equals $\frac{5}{3}$.

Figure 1 illustrates the concept of *support *and *weight-support*.

In order to make the support scores more comparable between drugs, we introduce normalized versions of the scores, *normalized support *and *normalized weighted support *which denote the respective support value divided the maximal possible *support *or *weighted support*, respectively.

### Odds ratio

For a given drug resistance profile *v *and mutation *p*, we calculate *odds ratio *using the formula:

(6) $$odds\text{\_}ratio_{v}\left(p\right)=\frac{n_{R1}\cdot n_{S0}}{max\left(1,n_{R0}\right)\cdot max\left(1,n_{S1}\right)}$$

Here, *n*_*R*1_, *n*_*S*0_, *n*_*R*0 _and *n*_*S*1 _denote the number of drug-resistant strains with mutation *p*, drug-susceptible strains without mutation *p*, drug-resistant strains without mutation *p *and drug-susceptible strains with mutation *p*, respectively.

The same formula is used to calculate odds ratio for gene gain/loss profiles.

### Statistical significance

In order to assess statistical significance of the associations we calculate their *p*-*value*.

More precisely, for a given drug resistance profile *v*, let *X *be the random variable giving *support *of a random mutation. Then, for a given observed mutation with *support *= *c*, its *p-value *is defined by the following formula:

(7) $$\mathbb{P}(X\geq c)=\sum_{n=1}^{\left|S\right|}\mathbb{P}(X\geq c|N=n)\cdot \mathbb{P}(N=n)$$

Here, *N *is a random variable which denotes the number of mutated strains in a random mutation. For each *n *the probability ℙ(*N *= *n*) of observing a mutation present in *n *strains is estimated (as the number of mutations present in *n *strains to the total number of considered mutations) from the data for point mutation and gene gain/loss profiles separately. The details follow. Assume that weights, for a given drug resistance profile *v*, take *k *different values: *l*_1_, *l*_2_, ..., *l_k_*. For 1 ≤ *j *≤ *k*, let *m_j _*be the number of strains which take value *l_j_*. Clearly we have *m*_1_ + *m*_2_ + ⋯ + *m*_*k*_ = |*S*|. Then, the probability ℙ(*X *≥ *c*|*N *= *n*) (from the equation 7) is given by the formula:

(8) $$\sum_{\begin{array}{l} \;\;\;\;\;\;\;0\leq n_{1}\leq m_{1}\\ \;\;\;\;\;\;0\leq n_{2}\leq m_{2}\\ \;\;\;\;\;\;\;\;\;\;\;\;\;\;\cdots \\ \;\;\;\;\;0\leq n_{k}\leq m_{k}\\ n_{1}+n_{2}+\cdots +n_{k}=n \end{array}}\frac{\prod_{j=1}^{k}{(}_{n_{j}}^{m_{j}})}{\left({}_{n}^{\left|S\right|}\right)}[\sum_{j=1}^{k}n_{j}\cdot \;l_{j}\geq c]$$

Here we describe our algorithm for calculating the p-value. It should be clear that the problem reduces to computing $\mathbb{P}\left(X\geq c|N=n\right)=\frac{t_{c}\left(n\right)}{\left(\begin{array}{c} \left|S\right|\\ n \end{array}\right)}$ for each 0 ≤ *n* ≤ |*S*|, where *t_c_*(*n*) denotes the number of ways for distributing *n *ones over |*S*| strains, such that the corresponding sum of weights is greater or equal than *c*. The term $\left(\begin{array}{c} \left|S\right|\\ n \end{array}\right)$ is the total number of possible ways for distributing *n *ones over |*S*| strains. Thus, the problem reduces to calculating *t_c_*(*n*) for each 0 ≤ *n* ≤ |*S*|. Additionally, without any loss of generality, we may assume that the weight levels are strictly decreasing: *l*_1 _>*l*_2 _> ⋯ >*l_k_*, where *l_k _*< 0 and *l*_*k*-1 _≥ 0.

The algorithm iteratively generates partial combinations (without *n_k_*) starting from the partial combination (*n*_1_ = *m*_1_, ⋯, *n*_*k* - 1_ = *m*_*k* - 1_) in the following manner: if *j *is the highest index of the non-zero *n_i _*in the current partial combination, the next partial combination will be (*n*_1_, ⋯, *n*_*j*_ - 1, *n*_*j* + 1_ = *m*_*j* + 1_, ⋯, *n*_*k* - 1_ = *m*_*k* - 1_). The algorithms terminates generating partial combinations when two following partial combinations have their corresponding sum of weights below the level of *c*. At each step of the algorithm, all possible full combinations (*n*_1_, ⋯ *n*_*k* - 1_, *n*_*k*_) are generated from the current partial combination (*n*_1_, ⋯ *n*_*k* - 1_). If for the full combination its corresponding sum of weights is greater or equal $c\left(\sum_{i=1}^{k}n_{i}\cdot l_{i}\geq c\right)$, then we increment the value *t_c_*(*n*) by $\prod_{j=1}^{k}\left(\begin{array}{c} m_{i}\\ n_{i} \end{array}\right)$, where *n* = *n*_1_ + ⋯ + *n*_*k*_. As the outcome, we obtain *t_c_*(*n*) and, thus, also ℙ(*X *≥ *c*|*N *= *n*) for each *n*.

The last step is to calculate formula 7 using these calculated probabilities.

Note that, since *support *is a special case of *weighted support*, the same formula and algorithm can be used to compute its corresponding p-values.

## Results and discussion

We verify the usability of our approach by trying to re-identify the known drug resistance determinants. In this experiment, we compare our proposed scoring methods --*support *and *weighted support *--to *odds ratio*, which is a popular measure used in genome-wide association studies. Table 1 shows rankings of the gene gain/loss profiles corresponding to genes which are known drug resistance determinants. The experiment suggests that *weighted support *identifies putative associations better than *support *and *odds ratio*, both of which do not incorporate additional information about phylogeny.

**Table 1:** Rankings of known drug resistance determining genes

		Rankings before prediction			Rankings after prediction		
gene id.	drug name	S	WS	OR	S	WS	OR
tet	Tetracycline	54.5	2.5	43.7	1.5	1.5	1.5
tetM	Tetracycline	14.5	11.5	7.5	4	4	4
mecA	Methicillin	1	1	1	1	1	1
mecA	Oxacillin	3	4	2	1	2	1
ermA1	Clindamycin	5.5	5.5	5.5	1	1	1
ermC	Clindamycin	907	471	907	414.5	11	191.5
ermA1	Erythromycin	3	3	4	1	1	1
ermC	Erythromycin	1527	3994.5	1006.5	413.5	28	214.5
aacA-aphD	Gentamicin	72	34	34	1	1	1
blaZ	Penicillin	163	66	223	1.5	1	2.5
mecA	Penicillin	163	8	223	11	5	52
Average ranking (excluding ermC):		53.27	**15.05**	60.411	2.55	**1.94**	7.22

Rankings of the known drug resistance determinants obtained by employing three different methods to score gene gain/loss profiles: *support *(S), *weighted support *(WS) and *odds ratio *(OR). Since some of the gene gain/loss profiles are assigned with the same score, we calculate their rankings as the arithmetic mean of positions of the profiles with the same score on the list sorted according to the scores; thus some of the rankings are not round numbers. The rankings were computed before and after prediction of drug resistance, which is based on the presence of the drug resistance determinants. We excluded the gene ermC from the calculations of the rankings since none of the methods were able to pull it out into the top 100 before prediction.

**Figure 2:**
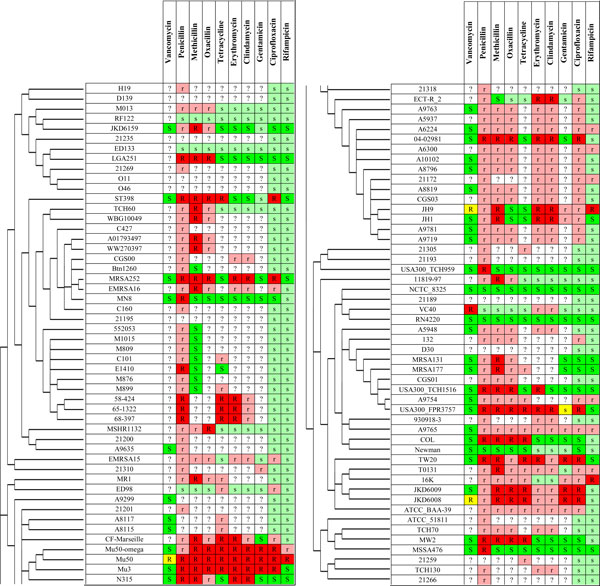
**The collected dataset of phenotypes with predictions**. The collected dataset of phenotypes put together with results of our drug resistance predictions based on the presence of known drug resistance determinants. Due to the high number of strains the table is split into two panels. Columns represent drugs, rows represent *S. aureus *strains included in the study in the order corresponding to the reconstructed phylogenetic tree of strains. Green, yellow and red cell colors represent susceptible, intermediate resistant and resistant phenotypes, respectively. Analogously, light green and light red cell colors represent predicted susceptible and resistant phenotypes, respectively. White cell color represents unknown (not determined by experiments or prediction) drug resistance phenotypes.

This experiment also reveals that the amount of the collected drug resistance information is not sufficient to correctly identify drug resistance associated genes. However, the high consistency of drug resistance profiles corresponding to the collected information and the presence of drug resistance determinants (summing over drugs, there are 117 drug resistant strains, where only 4 of them do not have any known drug resistance determinants; and there are 112 drug-susceptible strains, where only 8 of them have at least one drug resistance determinant) suggests that we can use the determinants to predict drug resistance in the strains without drug resistance information available. It is perhaps questionable to predict drug resistance in those strains for which the whole-genome sequence is not determined yet. So we do prediction only for those strains with completed sequencing or at least information on their plasmids (which often carry the drug resistance determinants). Nevertheless, we predict drug resistance also for those strains that are not yet fully sequenced, provided the presence of drug resistance determining genes has been confirmed for them. Moreover, we predict drug resistance to rifampicin and ciprofloxacin for all 100 strains, as the drug resistance for rifampicin and ciprofloxacin is determined by point mutations in genes *rpoB, gyrA *and *grlA *(synonymous name to *parC*), which are sequenced in all strains. More precisely, we predicted as rifampicin-resistant all strains with any mutation present in the rifampicin resistance determining region (RRDR). We defined the RRDR as the amino-acid range from 463 to 530 in the *rpoB *gene sequence (according to [94]). Analogously, we predicted as ciprofloxacin-resistant all strains with any point mutation in the quinolone resistance determining region (QRDR). We defined QRDR as the amino-acid ranges from position 68 to 107 and from position 64 to 103 in the *grlA *and *parC *gene sequences, respectively (according to [65]). Figure 2 shows the complete information about drug susceptibility after prediction.

Then, we applied our approach to the dataset supplemented by the predicted information about drug susceptibility for the following drugs: tetracycline, *β*-lactames (penicillin, oxacillin, methicillin), erythromycin, gentamicin, vancomycin, ciprofloxacin and rifampicin.

We discuss in the subsections below the results of our approach applied separately to the following drugs: tetracycline, *β*-lactames (penicillin, methicillin), erythromycin, gentamicin, vancomycin, ciprofloxacin. We do not discuss here results for oxacillin and clindamycin, since they have very similar drug resistance profiles to methicillin and erythromycin, respectively. All other drugs were excluded from the analysis due to the low number of strains with available drug resistance information on these drugs.

Tables 2 and 3 present the top-scored gene gain/loss, and point mutation profiles for the dis-cussed drugs, respectively. The genes presented in the tables were selected according to the following procedure: for each drug we construct a function, which gives for each gene (listed in descending order with respect to *normalized weighted support*) the minus logarithm of p-value (-*log*(p-value)) of this score. Then, we report genes which correspond to the portion of the graph of this function before it gets flattened. Complete results for all the drugs are provided in supplementary Excel tables (additional files 3 and 4).

**Table 2:** The top scored gene gain/loss profiles

Gene identifier	NS	NWS	OR	p-value	Gene functional annotation
**Penicillin (NWS-threshold: 0.58)**					

SAR1831(blaZ)	0.84	0.81	37.15	1.15e-06	beta-lactamase
SAR1829(blaI)	0.84	0.74	37.15	5.24e-06	transcriptional repressor
SAR1830(blaR1)	0.82	0.73	31.27	7.09e-06	beta-lactamase regulatory protein blar1
SAR0056	0.63	0.71	12.13	1.03e-05	conserved hypothetical protein
SAR0039(mecA)	0.61	0.70	10.94	1.28e-05	penicillin-binding protein pbp2a, methicillin resistance determinant mecA, transpeptidase
SAR0060(ccrA)	0.61	0.63	10.94	4.40e-05	resolvase, n-terminal domain protein
SAR0061(yycG)	0.61	0.63	10.94	4.40e-05	putative membrane protein
NWMN 0025	0.57	0.63	9.40	4.41e-05	conserved domain protein
SAR0037(ugpQ)	0.60	0.63	10.39	5.08e-05	glycerophosphoryldiester phosphodiesterase
SAR0038(maoC)	0.60	0.63	10.39	5.08e-05	dehydratase
SAR0057	0.57	0.59	9.40	9.78e-05	conserved hypothetical protein

Methicillin (NWS-threshold: 0.68)					

SAR0039(mecA)	1.00	1.00	950.00	4.48e-20	penicillin-binding protein pbp2a, methicillin resistance determinant mecA, transpeptidase
SAR0037(ugpQ)	0.98	0.94	931.00	6.77e-15	glycerophosphoryldiester phosphodiesterase
SAR0038(maoC)	0.98	0.94	931.00	6.77e-15	dehydratase
SAR0056	0.95	0.85	900.00	7.55e-12	conserved hypothetical protein
SAR0036	0.64	0.80	33.78	5.77e-11	putative membrane protein
SAR0057	0.85	0.75	162.00	6.47e-10	conserved hypothetical protein
SAR0060(ccrA)	0.91	0.73	432.00	1.40e-09	resolvase, n-terminal domain protein
SAR0061(yycG)	0.91	0.73	432.00	1.40e-09	putative membrane protein
MW0028(ebpS)	0.54	0.71	22.30	2.76e-09	hmg-coa synthase

Tetracycline (NWS-threshold: 0.32)					

SAAV_b3(repC)	0.54	0.64	27.69	5.70e-08	plasmid replication protein
SATW20_00660(tet)	0.54	0.64	27.69	5.70e-08	tetracycline resistance protein
SATW20_00670(pre)	0.50	0.50	24.00	3.51e-06	plasmid recombination enzyme type 3
SATW20_04620(tetM)	0.46	0.37	20.80	7.54e-05	tetracycline resistance protein tetM
SATW20_08990(virE)	0.42	0.37	19.93	7.67e-05	pathogenicity island protein
SATW20_09000	0.42	0.37	19.93	7.67e-05	pathogenicity island protein
SATW20_09010(lipA)	0.42	0.37	19.93	7.67e-05	putative protein in superantigen-encoding pathogenicity islands
SATW20_04610(thiI)	0.43	0.35	18.00	1.32e-04	putative transcriptional regulator
MW0745(int)	0.25	0.32	8.00	2.28e-04	site-specific recombinase, phage integrase family
MW0747	0.25	0.32	8.00	2.28e-04	DNA-binding helix-turn-helix protein

Erythromycin (NWS-threshold: 0.27)					

SAR0050(ermA1)	0.80	0.58	76.00	1.36e-06	rRNA adenine n-6-methyltransferase
CGSSa03 12660	0.47	0.44	17.19	2.98e-05	conserved hypothetical protein
SAR0054(tnpA1)	0.75	0.39	72.00	8.12e-05	transposase for transposon
SAR1734	0.75	0.39	72.00	8.12e-05	methylase
SAR1736(spc2)	0.75	0.39	72.00	8.12e-05	spectinomycin 9-o-adenylyltransferase
SaurJH9_1711(radC)	0.72	0.38	62.00	8.83e-05	predicted protein
SAUSA300_pUSA030006	0.20	0.35	4.75	1.65e-04	replication and maintenance protein
SAR1737(tnpC2)	0.72	0.34	62.00	1.89e-04	Unknown
SAR1529	0.33	0.33	9.15	2.43e-04	conserved hypothetical protein
SATW20_04860(recF_1)	0.23	0.30	5.52	3.67e-04	recombinational DNA repair ATPase
SAR1738(tnpB2)	0.70	0.29	54.00	4.39e-04	transposase B from transposon Tn554
SauraJ_010100009720	0.23	0.27	5.52	6.60e-04	conserved domain protein

Gentamicin (NWS-threshold: 0.83)					

SaurJH1_2806(aacA-aphD) SaurJH1_2805	0.83 0.75	0.90 0.83	150.00 90.00	9.38e-11 2.95e-09	bifunctional acetyltransferase/phosphotransferase GNAT family acetyltransferase

Ciprooxacin (NWS-threshold: 0.4)					

SATW20_04610(thiI)	0.35	0.45	36.00	1.33e-07	putative transcriptional regulator
SATW20_04650(cap8J)	0.32	0.40	31.57	8.25e-07	lipoprotein
SATW20_04670(capL)	0.32	0.40	31.57	8.25e-07	putative ATP/GTP-binding protein
SATW20_04780	0.32	0.40	31.57	8.25e-07	conjugation related protein
SATW20_04800	0.32	0.40	31.57	8.25e-07	replication initiation factor
SATW20_04810	0.32	0.40	31.57	8.25e-07	DNA segregation ATPase FtsK/SpoIIIE
SATW20_04830	0.32	0.40	31.57	8.25e-07	conjugative transposon protein

Summarizing information for the top scored gene gain/loss profiles. The consequent columns refer to: gene identifier of the corresponding gene family; *normalized support *(NS); *normalized weighted support *(NWS); *odds ratio *(OR); p-value and the gene functional annotation. Thresholds for *weighted support *are provided in brackets for each drug. Colored gene gain/loss profiles are provided in the supplementary the (additional file 3). Complete results for all the drugs are provided in the supplementary Excel table (additional files 2).

**Table 3:** The top scored point mutation profiles, only for essential mutations

Gene identifier	desc.	NS	NWS	OR	p-value	Gene functional annotation
**Penicillin (NWS-threshold: 0.4)**						

SAR0023(sasH)	G_723_D	0.55	0.63	8.51	1.87e-05	virulence-associated cell-wall-anchored protein; 5'-nucleotidase
SAR0023(sasH)	T_725_A	0.54	0.62	8.11	2.23e-05	virulence-associated cell-wall-anchored protein; 5'-nucleotidase
SAR0304	V_295_I	0.39	0.49	4.48	3.25e-04	acid phosphatase
SAR2791	V_182_M	0.46	0.46	6.05	5.41e-04	transcriptional regulator, Xre family
SAR2700	N_493_KD	0.52	0.45	7.72	6.16e-04	ABC transporter permease protein
SAR0233(hmp)	Q_333_K	0.44	0.44	5.48	7.21e-04	avohemoprotein (nitric oxide dioxygenase)
SAR0318(sbnA)	N_25_HK	0.44	0.43	5.48	8.36e-04	alpha/beta family hydrolase
SAR2664	V_282_AT	0.44	0.43	5.48	8.36e-04	probable monooxygenase
SAR2779	S_48_G	0.44	0.43	5.48	8.36e-04	n-hydroxyarylamine o-acetyltransferase
SAR0318(sbnA)	T_138_IM	0.43	0.43	5.21	8.36e-04	alpha/beta family hydrolase
SAR0318(sbnA)	T_139_AQ	0.43	0.43	5.21	8.36e-04	alpha/beta family hydrolase
SAR0023(sasH)	A_749_TG	0.41	0.43	4.96	8.44e-04	virulence-associated cell-wall-anchored protein; 5'-nucleotidase
SAR0318(sbnA)	R_130_CG	0.41	0.43	4.96	8.72e-04	alpha/beta family hydrolase
SAR0322(folC)	H_201_YQE	0.41	0.43	4.96	8.72e-04	macro domain, possibly adp-ribose binding module
SAR0233(hmp)	K_323_ET	0.40	0.42	4.71	9.08e-04	avohemoprotein (hemoglobin-like protein)
SAR2750(icaC)	I_21_V	0.40	0.42	4.71	9.46e-04	polysaccharide intercellular adhesin (PIA) biosynthesis protein
SAR0233(hmp)	S_309_RN	0.39	0.42	4.48	9.46e-04	avohemoprotein (hemoglobin-like protein)

Methicillin (NWS-threshold: 0.25)						

SAR0198(oppF)	T_287_IK	0.10	0.29	2.11	1.41e-04	putative glutathione transporter, ATP-binding component
SAR0420	I_72_F	0.10	0.29	2.11	1.41e-04	membrane protein
SAR2508(sbi)	S_219_AT	0.10	0.29	2.11	1.41e-04	IgG-binding protein Sbi
SAR2508(sbi)	N_222_QK	0.10	0.29	2.11	1.41e-04	IgG-binding protein Sbi
SAR2508(sbi)	K_224_SDN	0.10	0.29	2.11	1.41e-04	IgG-binding protein Sbi

Tetracycline (NWS-threshold: 0.2)						

SAR1840	D_291_YS	0.18	0.23	5.22	7.09e-04	NAD(FAD)-utilizing dehydrogenases
SAR2336(rpsJ)	K_57_M	0.29	0.23	9.60	7.32e-04	SSU ribosomal protein S10P (S20E)
SAR0550(rpsL)	K_113_R	0.36	0.20	13.33	1.14e-03	SSU ribosomal protein S12P (S23E)

Erythromycin (NWS-threshold: 0.2)						

SAR0576	A_68_EV	0.07	0.21	1.54	8.89e-04	phosphoglycolate phosphatase

Gentamicin (NWS-threshold: 0.21)						

SAR1840	L_289_IW	0.33	0.29	15.00	1.43e-03	NAD(FAD)-utilizing dehydrogenases
SAR1840	D_291_YS	0.33	0.29	15.00	1.43e-03	NAD(FAD)-utilizing dehydrogenases
SAR1840	H_327_RF	0.33	0.29	15.00	1.43e-03	NAD(FAD)-utilizing dehydrogenases
SAR1167(ylmH)	K_215_N	0.25	0.29	10.00	1.43e-03	RNA-binding S4 domain-containing protein
SAR1167(ylmH)	R_216_V	0.25	0.29	10.00	1.43e-03	RNA-binding S4 domain-containing protein
SAR1167(ylmH)	V_217_L	0.25	0.29	10.00	1.43e-03	RNA-binding S4 domain-containing protein
SAR0547(rpoB)	D_471_YG	0.17	0.21	6.00	4.61e-03	DNA-directed RNA polymerase beta subunit
SAR1833(trmB)	T_54_IK	0.17	0.21	6.00	4.61e-03	tRNA (guanine46-n7-)-methyltransferase

Ciprooxacin (NWS-threshold: 0.12)						

SAR1367(grlA)	S_80_YF	1.00	1.00	2244.00	6.03e-30	topoisomerase IV subunit a
SAR0006(gyrA)	S_90_AL	0.94	0.88	1056.00	1.92e-18	DNA gyrase subunit a
SAR2449(lytT)	V_45_I	0.21	0.20	17.11	2.06e-04	transcriptional regulator
SAR1840	L_289_IW	0.12	0.20	8.80	4.56e-04	NAD(FAD)-utilizing dehydrogenases
SAR1793(thiI)	A_92_ET	0.09	0.20	6.39	2.06e-04	thiamine biosynthesis protein thiI
SAR2212(murA2)	A_102_T	0.06	0.20	4.12	2.06e-04	UDP-n-acetylglucosamine 1-carboxyvinyltransferase
SAR1367(grlA)	E_84_KG	0.26	0.15	23.76	9.40e-04	topoisomerase IV subunit a
SAR0235(pstG 1)	F_401_LV	0.09	0.13	6.39	2.21e-03	PTS system, maltose and glucose-specific IIC component
SAR0400(nfrA)	R_194_H	0.09	0.13	6.39	2.21e-03	nitroreductase family protein

Summarizing information for the top scored point mutation profiles, only for essential mutations. The *conflict mutatations *were removed from the table for: tetracycline, erythromycin and gentamicin (for the rest of drugs there were no *conflict mutations *above the set thresholds). The consequent columns refer to: gene identifier of the corresponding gene family; corresponding position in the multiple alignment and changed amino acids; *normalized support *(NS); *normalized weighted support *(NWS); *odds ratio *(OR); p-value and the gene functional annotation. Thresholds for *weighted support *are provided in brackets for each drug. Colored gene gain/loss profiles are provided in the supplementary the (additional file 4). Complete results for all the drugs are provided in the supplementary Excel table (additional files 5).

### Tetracycline

Tetracycline acts by binding to the 30S ribosomal subunit (rpsS, 16S rRNA are its direct targets), preventing binding of tRNA to the mRNA-ribosome complex, and thus inhibiting protein synthesis [7].

The most common drug resistance mechanism to tetracycline in *S. aureus *is mediated by ribosome protection proteins (RPPs) such as *tet *and *tetM*, which bind to the ribosome complex, thus preventing the binding of tetracycline [99,100].

Proteins *tet *and *tetM *mediating the mechanism cover all drug-resistant strains except MW2. This may be caused by errors in the drug susceptibility tests, errors in sequencing, or by some other not yet known drug resistance mechanism. The inconsistent information about strain MW2's tetracyline susceptibility (see supporting Table 1) and the lack of identified drug resistance determinants suggest that the strain is possibly drug susceptible. In our experiment we initially assumed that the tetracycline resistance information is not available for strain MW2.

Our method shows that, besides *tet *and *tetM*, there are a few more genes that have highly scored gene gain/loss profiles. Especially interesting are the following genes which are not gained by any of the drug susceptible strains: *repC, pre, thiI, int, clfB *(see Table 2). There are studies reporting the significance of these *clfB *and *repC *genes in drug resistance [101,102]. Interestingly, the gene *repC *seems to co-evolve with *tet *(correlated gene gain/loss profiles).

Applying our method to point mutations we have identified two highly scored (and essential) point mutations in ribosomal complex proteins: *K*_101_*R *in *rpsL *and *K*_57_*M *in *rpsJ*. According to our knowledge, this is the first report on the significance of the point mutations for drug resistance in *S. aureus*. However, we found a study associating mutations in *rpsJ *with tetracycline resistance in another bacteria *Neisseria gonorrhoeae *[103].

### Beta-lactams

Beta-lactams are a broad class of antibiotics, which possess (by definition) the *β*-lactam ring in their structure. The ring is capable of binding transpeptidase proteins (also known as Penicillin Binding Proteins -- PBPs) [7], which are important to synthesis of the peptidoglycan layer of bacterial cell wall. PBPs with attached drug molecules are no longer able to synthesize peptidoglycan, leading to bacterial death [104]. In our case study we consider three *β*-lactam antibiotics: penicillin, oxacillin and methicillin. However, since the drug resistance profile and drug resistance mechanisms for oxacillin and methicillin are very similar we discuss results only for methicillin.

There are two common *β*-lactames resistance mechanisms in *S. aureus *[104,105]. The first one is mediated by *β*-lactamase enzymes, which bind drug molecules and break the *β*-lactam ring, thus deactivating the drug molecules. This mechanism is effective against penicillin (which is *β*-lactamase sensitive) and not effective against methicillin and oxacillin (which are *β*-lactamase resistant) [106]. The second *β*-lactam resistance mechanism is mediated by proteins which are capable of functionally substituting for PBPs, but have much smaller affinity to *β*-lactam molecules. This mechanism is effective against penicillin, methicillin and oxacillin.

#### Penicillin

In our dataset all strains resistant to penicillin possess proteins responsible for one of the two mechanisms. More precisely, there are 69 drug-resistant strains (with available drug resistance information), which possess *BlaZ *-- the standard *β*-lactamase protein (note that its regulators *BlaR1 *and *blaI *do not always co-occur). All the remaining penicillin-resistant strains have *mecA*, which is an altered PBP. Table 2 provides information about the top-scored gene gain/loss profiles.

Applying our method we have also identified the uncategorized putative protein, *SAR0056*, as putatively associated with penicillin resistance (see Table 2). We suggest to examine further the role of that gene in *β*-lactams resistance.

#### Methicillin

**Figure 3:**
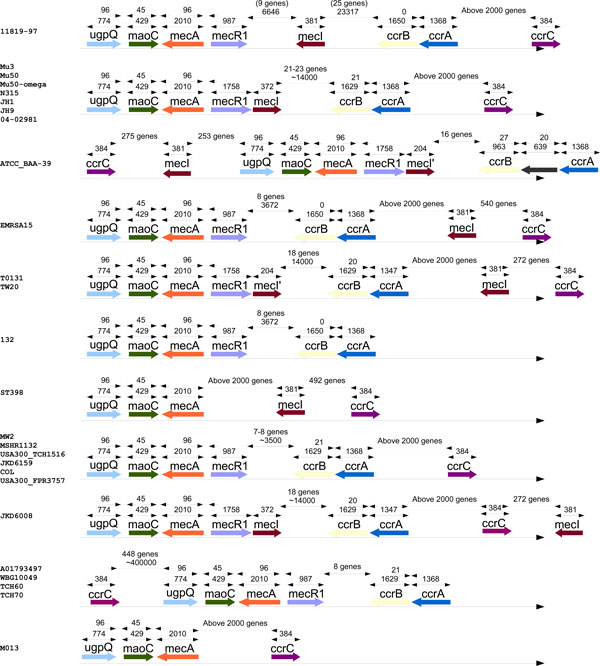
**Genes related to methicillin resistance**. Presence and relative genome coordinates of genes related to methicillin resistance (*mecA, mecR1, mecI, ccrA, ccrB, ccrC*), put together with the identified genes: *ugpQ *and *maoC*. The gene presence profiles are clustered with respect to the genes order. In this figure we include only these methicillin-resistant strains for which all the genes where located on the main genome and within the same sequence conting (in order to determine the relative positions).

Applying our approach to gene gain/loss profiles we identified (beside *mecA*) genes *ugpQ *and *maoC*. The correlation of gene profiles to the profile of *mecA *and their close proximity on the genomes suggests that these genes co-evolve (see Figure 3 for more details). This co-evolution may reflect some important role played by these genes in methicillin resistance. This calls for further study of the role of these two genes in methicillin resistance.

We have also identified a few point mutations that are putatively associated with methicillin resistance. Interestingly, two of the mutations in the top 10 essential mutations according to *weighted support *(*I*_72_*F *in *SAR0420 *and *E*_208_*QKD *in *SAR0436*) are present in cell membrane proteins. This suggests some compensatory mechanism to the presence of *mecA*.

### Ciprofloxacin

Ciprofloxacin belongs to a broad class of antibiotics, called fluoroquinolones, which are functional against bacteria by binding DNA gyrase subunit A (encoded by *gyrA*) and DNA topoisomerase 4 subunit A (encoded by *parC*), which are enzymes necessary to separate bacterial DNA, thereby inhibiting cell division [7]. The most common ciprofloxacin resistance mechanism is mediated by point mutations in the drug targets, *parC *and *gyrA*.

Applying our approach we identified (by highest support) two point mutations in ciprofloxacin target genes -- *S*_80 _*FY *in *parC *and *S*_90 _*AL *in *gyrA *-- which are located in QRDR and known to be responsible for the first mechanism of ciprofloxacin resistance [65]. The presence of these mutations is correlated with the ciprofloxacin resistance profile for strains with available drug resistance information. However, they differ for two strains ED98 and 16 K (only the mutation in *parC *is present). This may suggest intermediate drug resistance level for these strains. Unfortunately ciprofloxacin resistance information is not available for these strains.

### Erythromycin

Erythromycin acts by binding the 23S rRNA molecule (in the 50S subunit) of the bacterial ribosome complex, leading to inhibition of protein synthesis [7].

There are three known erythromycin resistance mechanisms [107]. First -- the most common mechanism -- is by methylation (addition of two residues to the domain V of 23S rRNA) of the 23S rRNA molecule, which prevents the ribosome from binding with erythromycin. This methylation is mediated by enzymes from the *erm *gene family, the most common are *ermA *and *ermC*. The second mechanism is mediated by the presence of macrolide efflux pumps (encoded by *msrA *and *msrB*). The third mechanism is the inactivation of drug molecules by specialized enzymes such as *EreA *or *EreB *[107].

We found that none of the strains in our case study possess genes *EreA *or *EreB*. Genes encoding efflux pumps (*msrA *and *msrB*) are present also in drug-susceptible strains (for example, NCTC 8325 and Newman), which may suggest that the mechanism is inactive for the considered strains of *S. aureus *or the enzyme production rates are too small, which we are not able to account by our method. Using our approach we identified (by the highest support) the gene *ermA *responsible for the most common drug resistance mechanism.

Here, there is one erythromycin-susceptible strain, USA300_TCH959, which harbours the *ermA *gene. This may suggest disruption of the drug resistance mechanism in that strain, errors in drug susceptibility testing or errors in sequencing.

Interestingly, we identified gene *SAR1736(spc2) *(which is a known spectinomycin resistance determinant) as potentially associated with erythromycin resistance. This suggests that drug resistance to spectinomycin and erythromycin co-evolved, despite these two drugs belonging to different classes according to the ATC drug classification system [106].

### Gentamicin

Gentamicin works by inhibition of protein synthesis by binding the 30S subunit of the ribosome complex [108].

Interestingly, strain USA300-FPR3757 exhibits intermediate drug resistance, which is correlated with the absence of *aacA-aphD *gene in its genome sequence. Since our method requires binary information on drug susceptibility, we marked this strain as drug susceptible for experiments.

The most common resistance mechanism responsible for high levels of Gentamicin resistance is mediated by the drug-modifying enzyme *SaurJH1_2806(aacA-aphD)*. Applying our methodology we identified the gene encoding it as likely to be associated with drug resistance (maximal support). Moreover, we identified also the gene *SaurJH1_2805 *as putatively associated with gentamicin resistance. The close proximity of these two genes in the genomes and their highly correlated gene gain/loss profiles suggest co-evolution. We hypothesize that the gene *SaurJH1_2805 *plays some role in drug resistance for gentamicin.

## Conclusion

In this work we present a novel approach to associate genes and mutations with drug resistance phenotypes by comparative analysis of fully sequenced bacterial strains (within the same species).

In order to apply our approach we collected genotype and phenotype data. Genome sequences and annotations were downloaded for 100 fully sequenced *S. aureus *strains. A challenge was to collect drug resistance information, which is spread throughout the literature. We retrieved the data from 71 publications. The collected dataset is available in the supplementary material (additional file 1).

In our method we consider two types of genetic differences as potentially associated with drug resistance: nonsynonymous point mutations and gene acquisition, represented by their mutation and gene gain/loss profiles, respectively. Then, the approach is based on the newly introduced concept of *support*, which is a score assigned to all mutation and gene gain/loss profiles. Intuitively, the higher support of a mutation profile, the better chance of the mutation to be associated with the drug-resistant phenotype. We also generalize the concept of support into *weighted support*, which incorporates phylogenetic information.

Applying our approach, we were able to successfully re-identify most of the known drug resistance determinants. Here, on average, *weighted support *outperforms *support *and *odds ratio*.

Moreover, applying our methodology, we identified some putative novel resistance-associated genes and mutations. We expect that these associations and drug resistance predictions will attract the experimental research community to verify their role in drug resistance mechanisms.

Finally, although the presented approach shows promise, it has some obvious limitations. Firstly, it is not clear what threshold for the *weighted support *is appropriate. Secondly, in this approach we only consider genome variations, whereas some drug resistance mechanisms may be related to changes in protein production rates (such as efflux pumps). Thirdly, the current approach ignores the role of non-coding RNA in drug resistance mechanisms. That, would not be detected by our approach. We plan to address these mentioned problems in some future work.

## Competing interests

The authors declare that they have no competing interests.

## Authors' contributions

All authors contributed to the design of the method, analysis of results and writing of the manuscript. MW wrote software and performed experiments. All authors read and approved the final manuscript.

## Supplementary Material

Additional file 1 **Collected phenotype and genotype data**. Collected dataset of phenotypes (results of drug susceptibility tests). Columns represent drugs, rows represent *S. aureus *strains included in the study, put in the order corresponding to the reconstructed phylogenetical tree of strains. Green, yellow and red color cells represent collected information: susceptibility, intermediate resistance and resistance of isolates, respectively. Click here for file

Additional file 4 **Excel table with detailed results for point mutation profiles**. Excel table providing results of our approach applied to point mutation profiles for ten drugs: penicillin, methicillin, oxacillin, tetracycline, clindamycin, erythromycin, gentamicin, ciprofloxacin, rifampicin and vancomycin Click here for file

Additional file 2 **Summary table for the top scored gene gain/loss profiles (same thresholds as for Table 2 are applied). **The columns refer to: gene identifier of the corresponding gene family; normalized *weighted support* (NWS); p-value and the drug resistance profiles put together with gene gain/loss profiles. Each cell in the gene gain/loss profiles corresponds to one strain, ordered according to the order in Figure 2. Cells corresponding to drug-resistant and drug-susceptible strains are colored red and green, respectively. Strains without drug resistance information are left white. For each gene gain/loss profile p and its corresponding row, if a cell in this row corresponds to strain *i*, such that *rv(p)* = *p(i)*, then it is colored blue, otherwise it is colored pink. Click here for file

Additional file 3 **Excel table with detailed results for gene gain/loss profiles**. Excel table providing results of our approach applied to gene gain/loss profiles for ten drugs: penicillin, methicillin, oxacillin, tetracycline, clindamycin, erythromycin, gentamicin, ciprofloxacin, rifampicin and vancomycin. Click here for file

Additional file 5 **Table with point mutation profiles for top scored mutations profiles**. Summary table for the top scored gene point mutation profiles (same thresholds as for Table 3 are applied). The columns refer to: gene identifier of the corresponding gene family; *normalized weighted support *(NWS); p-value and the drug resistance profiles put together with point mutation profiles. Each cell in the point profiles corresponds to one strain, ordered according to the order in Figure 2. Cells corresponding to drug-resistant and drug-susceptible strains are colored red and green, respectively. Strains without drug resistance information are left white. For each point mutation profile *p *and its corresponding row, if a cell in this row corresponds to strain *i *(assuming the corresponding gene is present in the strain sequence), such that *r_v_*(*p*) = *p*(*i*), then it is colored blue, otherwise it is colored pink. Cells corresponding to strains without the corresponding gene are left white. Click here for file
