# Supplementary material for: An approach to identifying drug resistance associated mutations in bacterial strains
Source: BMC Genomics. 2012 Dec 7;13(Suppl 7):S23. doi: 10.1186/1471-2164-13-S7-S23 (PMC3521396; doi:10.1186/1471-2164-13-S7-S23)

Gene identifier	NWS	p-value	Drug resistance and gene gain/loss profiles
Penicillin (NWS-threshold: 0.62)			
SAR1831(blaZ)	0.81	1.15e-06	
SAR1829(blaI)	0.74	5.24e-06	
SAR1830(blaR1)	0.73	7.09e-06	
SAR0056	0.71	1.03e-05	
SAR0039(mecA)	0.70	1.28e-05	
SAR0060(ccrA)	0.63	4.40e-05	
SAR0061(yycG)	0.63	4.40e-05	
NWMN_0025	0.63	4.41e-05	
SAR0037(ugpQ)	0.63	5.08e-05	
SAR0038(maoC)	0.63	5.08e-05	
Meticillin (NWS-threshold: 0.68)			
SAR0039(mecA)	1.00	4.48e-20	
SAR0037(ugpQ)	0.94	6.77e-15	
SAR0038(maoC)	0.94	6.77e-15	
SAR0056	0.85	7.55e-12	
SAR0036	0.80	5.77e-11	
SAR0057	0.75	6.47e-10	
SAR0060(ccrA)	0.73	1.40e-09	
SAR0061(yycG)	0.73	1.40e-09	
MW0028(ebpS)	0.71	2.76e-09	
Tetracycline (NWS-threshold: 0.37)			
SAAV_b3(repC)	0.64	5.70e-08	
SATW20_00660(tet)	0.64	5.70e-08	
SATW20_00670(pre)	0.50	3.51e-06	
SATW20_04620(tetM)	0.37	7.54e-05	
SATW20_08990(virE)	0.37	7.67e-05	
SATW20_09000	0.37	7.67e-05	
SATW20_09010(lipA)	0.37	7.67e-05	
Erythromycin (NWS-threshold: 0.39)			
SAR0050(ermA1)	0.58	1.36e-06	
CGSSa03_12660	0.44	2.98e-05	
Gentamicin (NWS-threshold: 0.83)			
SaurJH1_2806(aacA-aphD)	0.90	9.38e-11	
SaurJH1_2805	0.83	2.95e-09	
Ciprofloxacin (NWS-threshold: 0.45)			
SATW20_04610(thiI)	0.45	1.33e-07	

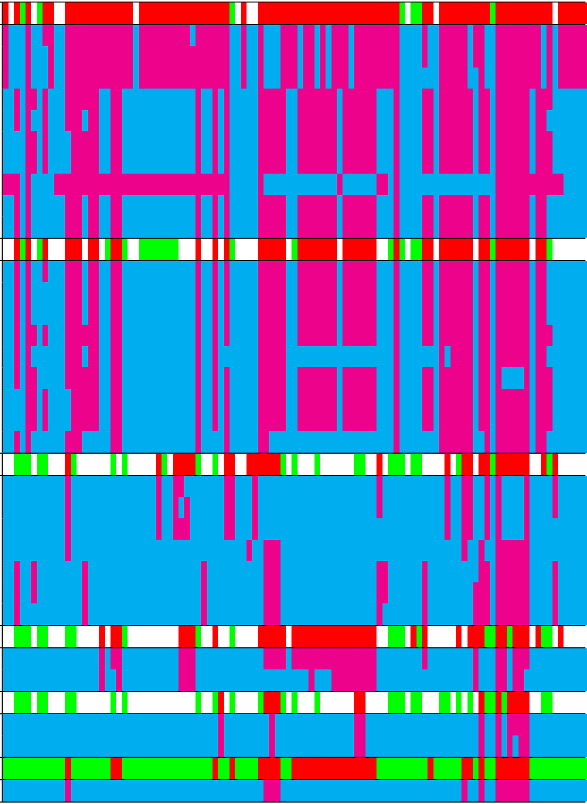

Supplement: Additional file 2 — Summary table for the top scored gene gain/loss profiles (same thresholds as for Table 2 are applied). The columns refer to: gene identifier of the corresponding gene family; normalized weighted support (NWS); p-value and the drug resistance profiles put together with gene gain/loss profiles. Each cell in the gene gain/loss profiles corresponds to one strain, ordered according to the order in Figure 2. Cells corresponding to drug-resistant and drug-susceptible strains are colored red and green, respectively. Strains without drug resistance information are left white. For each gene gain/loss profile p and its corresponding row, if a cell in this row corresponds to strain i, such that rv(p) = p(i), then it is colored blue, otherwise it is colored pink. [file 1471-2164-13-S7-S23-S2.pdf]
