# Supplementary material for: An approach to identifying drug resistance associated mutations in bacterial strains
Source: BMC Genomics. 2012 Dec 7;13(Suppl 7):S23. doi: 10.1186/1471-2164-13-S7-S23 (PMC3521396; doi:10.1186/1471-2164-13-S7-S23)

Gene identifier	desc.	NWS	p-value	Drug resistance and point mutation profiles
Penicillin (NWS-threshold: 0.4)				
SAR0023(sasH)	$G_{723}D$	0.63	1.87e-05	
SAR0023(sasH)	$T_{725}A$	0.62	2.23e-05	
SAR0304	$V_{295}I$	0.49	3.25e-04	
SAR2791	$V_{182}M$	0.46	5.41e-04	
SAR2700	$N_{493}KD$	0.45	6.16e-04	
SAR0233(hmp)	$Q_{333}K$	0.44	7.21e-04	
SAR0318(sbnA)	$N_{25}HK$	0.43	8.36e-04	
SAR2664	$V_{282}AT$	0.43	8.36e-04	
SAR2779	$S_{48}G$	0.43	8.36e-04	
SAR0318(sbnA)	$T_{138}IM$	0.43	8.36e-04	
SAR0318(sbnA)	$T_{139}AQ$	0.43	8.36e-04	
SAR0023(sasH)	$A_{749}TG$	0.43	8.44e-04	
SAR0318(sbnA)	$R_{130}CG$	0.43	8.72e-04	
SAR0322(folC)	$H_{201}YQE$	0.43	8.72e-04	
SAR0233(hmp)	$K_{323}ET$	0.42	9.08e-04	
SAR2750(icaC)	$I_{21}V$	0.42	9.46e-04	
SAR0233(hmp)	$S_{309}RN$	0.42	9.46e-04	
Meticillin (NWS-threshold: 0.25)				
SAR0198(oppF)	$T_{287}IK$	0.29	1.41e-04	
SAR0420	$I_{72}F$	0.29	1.41e-04	
SAR2508(sbi)	$S_{219}AT$	0.29	1.41e-04	
SAR2508(sbi)	$N_{222}QK$	0.29	1.41e-04	
SAR2508(sbi)	$K_{224}SDN$	0.29	1.41e-04	
Tetracycline (NWS-threshold: 0.2)				
SAR1840	$D_{291}YS$	0.23	7.09e-04	
SAR2336(rpsJ)	$K_{57}M$	0.23	7.32e-04	
SAR0550(rpsL)	$K_{113}R$	0.20	1.14e-03	
Erythromycin (NWS-threshold: 0.2)				
SAR0576	$A_{68}EV$	0.21	8.89e-04	
Gentamicin (NWS-threshold: 0.21)				
SAR1840	$L_{289}IW$	0.29	1.43e-03	
SAR1840	$D_{291}YS$	0.29	1.43e-03	
SAR1840	$H_{327}RF$	0.29	1.43e-03	
SAR1167(ylmH)	$K_{215}N$	0.29	1.43e-03	
SAR1167(ylmH)	$R_{216}V$	0.29	1.43e-03	
SAR1167(ylmH)	$V_{217}L$	0.29	1.43e-03	
SAR0547(rpoB)	$D_{471}YG$	0.21	4.61e-03	
SAR1833(trmB)	$T_{54}IK$	0.21	4.61e-03	
Ciprofloxacin (NWS-threshold: 0.2)				
SAR1367(grlA)	$S_{80}YF$	1.00	6.03e-30	
SAR0006(gyrA)	$S_{90}AL$	0.88	1.92e-18	
SAR2449(lytT)	$V_{45}I$	0.20	2.06e-04	
SAR1840	$L_{289}IW$	0.20	4.56e-04	
SAR1793(thiI)	$A_{92}ET$	0.20	2.06e-04	
SAR2212(murA2)	$A_{102}T$	0.20	2.06e-04	

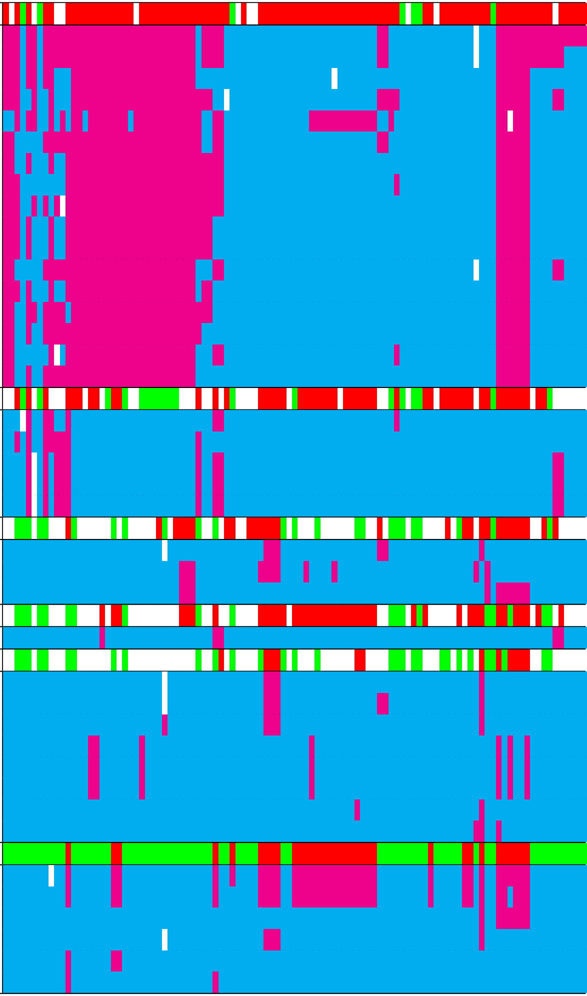

Supplement: Additional file 5 — Table with point mutation profiles for top scored mutations profiles. Summary table for the top scored gene point mutation profiles (same thresholds as for Table 3 are applied). The columns refer to: gene identifier of the corresponding gene family; normalized weighted support (NWS); p-value and the drug resistance profiles put together with point mutation profiles. Each cell in the point profiles corresponds to one strain, ordered according to the order in Figure 2. Cells corresponding to drug-resistant and drug-susceptible strains are colored red and green, respectively. Strains without drug resistance information are left white. For each point mutation profile p and its corresponding row, if a cell in this row corresponds to strain i (assuming the corresponding gene is present in the strain sequence), such that rv(p) = p(i), then it is colored blue, otherwise it is colored pink. Cells corresponding to strains without the corresponding gene are left white. [file 1471-2164-13-S7-S23-S5.pdf]
