# Supplementary material for: An approach to identifying drug resistance associated mutations in bacterial strains
Source: BMC Genomics. 2012 Dec 7;13(Suppl 7):S23. doi: 10.1186/1471-2164-13-S7-S23 (PMC3521396; doi:10.1186/1471-2164-13-S7-S23)
Supplement: Additional file 1 — Collected phenotype and genotype data. Collected dataset of phenotypes (results of drug susceptibility tests). Columns represent drugs, rows represent S. aureus strains included in the study, put in the order corresponding to the reconstructed phylogenetical tree of strains. Green, yellow and red color cells represent collected information: susceptibility, intermediate resistance and resistance of isolates, respectively. [file 1471-2164-13-S7-S23-S1.pdf]

Table 1. Complete collected data on drug resistance.

Strain name	Mupirocin	Doxycycline	Tetracycline	Minocycline	Tigecycline	Chloramphenicol	Ampicillin	Amoxicillin-clavulanat	Penicillin	Meticillin	Oxacillin	Flucloxacillin	Cefalexin	Cefazolin	Cefoxitin	Cefotaxime	Ceftriaxone	Ceftizoxime
	D	J																
	D06	J01																
	D06A	J01A				J01B	J01C						J01D					
	D06AX	J01AA				J01BA	J01CA		J01CE	J01CF			J01DB		J01DC	J01DD		
RF122																		
M013																		
O11																		
O46																		
21269																		
ED133																		
LGA251	S [21]		S [21]			S [21]			R [21]	R [21]	R : 16 [21]				R : 32 [21]			
21235																		
H19																		
D139																		
A9635											S : 0.25 [54]							
21200																		
ST398			R [60]						R [62]	R [60]	R [62]							
TCH60										R [44]								
WBG10049										R [56]								
21195																		
C427																		
C160																		
A01793497																		
WW270397																		
MRSA252			S [27]						R :> 2 [18]R [27]	R [27]	R :> 16 [18]							
EMRSA16										R [56]								
MN8			S : 0.12 [14]						R :> 2 [18]	S [40]	S : 0.12 [18]							
Btn1260										S [56]								
CGS00																		
E1410			S [9]						R [9]	S [56]								
552053																		
[illegible]	[illegible]	[illegible]	[illegible]	[illegible]	[illegible]	[illegible]	[illegible]	[illegible]	[illegible]	[illegible]	[illegible]	[illegible]	[illegible]	[illegible]	[illegible]	[illegible]	[illegible]	[illegible]
JH1			S [45]							R [45]	S : 0.75 [45]							
A9719																		
A9781																		
A8117																		
A8115																		
N315			S : ≤ 4 [2]			S : 8 [10]	U : 8 [2]	U :> 4 [2]	R :> 2 [18]R :> 8 [2]	R : 4 [35]R [35]	R :> 16 [18]R :> 2 [2]R : 8 [36]S : 2 [47]		U : 64 [36]	R :> 16 [2]		R :> 256 [2]	R :> 32 [2]	[illegible]
Mu3			R [67]			I [67]			R [67]	R [47]	R [67]R : 1025 [47]							
Mu50	S : 0.25 [43]S : 0.032 [51]	I : 8 [11]	R [67]R : 128 [25]R :> 32 [11]R : 32 [18]R : 64 [43]	I : 12 [11]		S : 4 [11]S [67]	U : 64 [25]U : 8 [11]		R [67]R :> 8 [18]	R : 512 [35]R :> 256 [11]	R :> 32 [18]R [67]R : 512 [43]R : 512 [47]R :> 256 [11]				R :> 256 [11]		R :> 256 [11]	[illegible]
Mu50-omega		I : 8 [11]	R :> 32 [11]	I : 10 [11]		S : 4 [11]	U : 1 [11]			R :> 256 [11]	R :> 8 [11]				R : 32 [11]		I : 3 [11]	[illegible]

Strain name	Ertapenem	Imipenem	Trimethoprim	Sulfamethoxazole	Co-trimoxazole	Erythromycin	Spiramycin	Clarithromycin	Azithromycin	Clindamycin	Lincomycin	Quinupristin/dalfopristin	Streptomycin	Tobramycin	Gentamicin	Kanamycin	Neomycin	Amikacin	Netilmicin	Sisomicin
	J01D		J01E			J01F							J01G							
	J01DH		J01EA	J01EC	J01EE	J01FA				J01FF		J01FG	J01GA	J01GB						
RF122																				
M013																				
O11																				
O46																				
21269																				
ED133																				
LGA251			S [21]			S [21]				S [21]					S [21]		S [21]			
21235																				
H19																				
D139																				
A9635																				
[illegible]	[illegible]	[illegible]	[illegible]	[illegible]	[illegible]	[illegible]	[illegible]	[illegible]	[illegible]	[illegible]	[illegible]	[illegible]	[illegible]	[illegible]	[illegible]	[illegible]	[illegible]	[illegible]	[illegible]	[illegible]

Strain name	Ofloxacin	Ciprofloxacin	Enoxacin	Temafloxacin	Norfloxacin	Lomefloxacin	Fleroxacin	Sparfloxacin	Levofloxacin	Trovafloxacin	Moxifloxacin	Gatifloxacin	Pazufloxacin	Vancomycin	Teicoplanin	Polymyxin	Fusidic acid	Nitrofurantoin	Fosfomycin	Spectinomycin	Linezolid	Daptomycin	Bacitracin	D-Cycloserine	Rifampicin	Clofazimine	Gramicidin
	J01																							J04			R
	J01M													J01X										J04A		J04B	R02
	J01MA													J01XA		J01XB	J01XC	J01XE	J01XX					J04AB		J04BA	R02AB
RF122																				R : 64 [24]							
M013																											
O11																											
O46																											
21269																											
ED133																											
LGA251		S [21]													S [21]		S [21]				S [21]				S [21]		
21235																											
H19																											
D139																											
A9635														S : 1 [54]								S : 0.5 [54]					
[illegible]	[illegible]	[illegible]	[illegible]	[illegible]	[illegible]	[illegible]	[illegible]	[illegible]	[illegible]	[illegible]	[illegible]	[illegible]	[illegible]	[illegible]	[illegible]	[illegible]	[illegible]	[illegible]	[illegible]	[illegible]	[illegible]	[illegible]	[illegible]	[illegible]	[illegible]	[illegible]	[illegible]

Comments

- legend: red: drug resistant; green: drug susceptible; yellow: intermediate drug resistant; gray: contrary information of no CLSI interpretation; white: not information is found,
- drug susceptibility information for the strain TW20 was confirmed by e-mail correspondence with the authors of [16]
- drug susceptibility information for the strain ST398 was confirmed by e-mail correspondence with the authors of [60]
- accordingly to [24] 'ET3-1 (RF122) is susceptible to most antibiotics, excluding Spectinomycin', according to [45] JH1 is susceptible to Rifampicin, with MIC 0.012 $\mu g/ml$. We use information from [45], treating it as more reliable, because this study is newer and provides the exact MIC value.

References

1. Tadashi Baba, Taeok Bae, Olaf Schneewind, Fumihiko Takeuchi, and Keiichi Hiramatsu. Genome sequence of staphylococcus aureus strain newman and comparative analysis of staphylococcal genomes: Polymorphism and evolution of two major pathogenicity islands. *Journal of Bacteriology*, 190(1):300–310, January 2008.
2. Amanda M. Beltramini, Chitrangada D. Mukhopadhyay, and Vijay Pancholi. Modulation of cell wall structure and antimicrobial susceptibility by a staphylococcus aureus Eukaryote-Like Serine/Threonine kinase and phosphatase. *Infection and Immunity*, 77(4):1406–1416, April 2009. PMID: 19188361 PMCID: 2663143.
3. Tamar Bikels-Goshen, Elad Landau, Sam Saguy, and Roni Shapira. Staphylococcal strains adapted to epigallocathechin gallate (EGCG) show reduced susceptibility to vancomycin, oxacillin and ampicillin, increased heat tolerance, and altered cell morphology. *International Journal of Food Microbiology*, 138(1–2):26–31, March 2010.
4. Gilles Brackman, Paul Cos, Louis Maes, Hans J. Nelis, and Tom Coenye. Quorum sensing inhibitors increase the susceptibility of bacterial biofilms to antibiotics in vitro and in vivo. *Antimicrobial Agents and Chemotherapy*, 55(6):26552661, 2011.
5. Kellie Burnside, Annalisa Lembo, Melissa de los Reyes, Anton Iliuk, Nguyen-Thao BinhTran, James E. Connelly, Wan-Jung Lin, Byron Z. Schmidt, Anthony R. Richardson, Ferric C. Fang, Weiguo Andy Tao, and Lakshmi Rajagopal. Regulation of hemolysin expression and virulence of \textitStaphylococcus aureus by a Serine/Threonine kinase and phosphatase. *PLoS ONE*, 5(6):e11071, 2010.
6. Kyra Chua, Torsten Seemann, Paul F Harrison, John K Davies, Scott J Coutts, Honglei Chen, Volker Haring, Robert Moore, Benjamin P Howden, and Timothy P Stinear. Complete genome sequence of staphylococcus aureus strain JKD6159, a unique australian clone of ST93-IV community Methicillin-Resistant staphylococcus aureus. *Journal of Bacteriology*, 192(20):5556–5557, October 2010.
7. Kyra Y. L. Chua, Torsten Seemann, Paul F. Harrison, Shaun Monagle, Tony M. Korman, Paul D. R. Johnson, Geoffrey W. Coombs, Brian O. Howden, John K. Davies, Benjamin P. Howden, and Timothy P. Stinear. The dominant australian Community-Acquired Methicillin-Resistant staphylococcus aureus clone ST93-IV [2B] is highly virulent and genetically distinct. *PLoS ONE*, 6(10):e25887, October 2011.
8. D. C Coleman, H. Pomeroy, J. K Estridge, C. T Keane, M. T Cafferkey, R. Hone, and T. J Foster. Susceptibility to antimicrobial agents and analysis of plasmids in gentamicin- and Methicillin-Resistant staphylococcus aureus from dublin hospitals. *Journal of Medical Microbiology*, 20(2):157–167, October 1985.
9. M. Ins Crisostomo, Henrik Westh, Alexander Tomasz, Marilyn Chung, Duarte C. Oliveira, and Herminia de Lencastre. The evolution of methicillin resistance in staphylococcus aureus: Similarity of genetic backgrounds in historically early methicillin-susceptible and -resistant isolates and contemporary epidemic clones. *Proceedings of the National Academy of Sciences of the United States of America*, 98(17):9865–9870, August 2001. PMID: 11481426 PMCID: 55544.
10. Kent B. Crossley, Kimberly K. Jefferson, Gordon L. Archer, and Vance G. Fowler. *Staphylococci in Human Disease*. John Wiley & Sons, November 2009.
11. Longzhu Cui, Hui-min Neoh, Mitsutaka Shoji, and Keiichi Hiramatsu. Contribution of vraSR and graSR point mutations to vancomycin resistance in Vancomycin-Intermediate staphylococcus aureus. *Antimicrobial Agents and Chemotherapy*, 53(3):1231–1234, March 2009.
12. Claire Daurel, Corinne Huet, Anne Dhalluin, Michle Bes, Jerome Etienne, and Roland Leclercq. Differences in potential for selection of Clindamycin-Resistant mutants between inducible erm(A) and erm(C) staphylococcus aureus genes. *Journal of Clinical Microbiology*, 46(2):546–550, February 2008.
13. Andrew P. Desbois and Peter J. Coote. Wax moth larva (\textitGalleria mellonella): an in vivo model for assessing the efficacy of antistaphylococcal agents. *Journal of Antimicrobial Chemotherapy*, 66(8):17851790, 2011.
14. N Dickgiesser and U Wallach. Toxic shock syndrome toxin-1 (TSST-1): influence of its production by subinhibitory antibiotic concentrations. *Infection*, 15(5):351–353, 1987. PMID: 3692606.
15. Binh An Diep, Steven R Gill, Richard F Chang, Tiffany HaiVan Phan, Jason H Chen, Matthew G Davidson, Felice Lin, Jessica Lin, Heather A Carleton, Emmanuel F Mongodin, George F Sensabaugh, and Franoise Perdreau-Remington. Complete genome sequence of USA300, an epidemic clone of community-acquired meticillin-resistant staphylococcus aureus. *The Lancet*, 367(9512):731–739, March 2006.
16. Jonathan D Edgeworth, Ghasem Yadegarfar, Smriti Pathak, Rahul Batra, Joshua D Cockfield, Duncan Wyncoll, Richard Beale, and di A Lindsay. An outbreak in an intensive care unit of a strain of MethicillinResistant \textitStaphylococcus aureus sequence type 239 associated with an increased rate of vascular access DeviceRelated bacteremia. *Clinical Infectious Diseases*, 44(4):493501, February 2007.
17. M.B. Ekkelenkamp, M. Sekkat, N. Carpaij, A. Troelstra, and M.J. Bonten. Endocarditis due to meticillinresistant \textitStaphylococcus aureus originating from pigs. *Nederlands Tijdschrift Geneeskunde*, 150(44):24427, November 2006.
18. Lakshmi Eyyunni, Richard Pettyjohn, and Daniel F. Sahm. Network on antimicrobial resistance in staphylococcus aureus. http://www.narsa.net/, 2011.
19. L Ferrero, B Cameron, and J Crouzet. Analysis of gyrA and grlA mutations in stepwise-selected ciprofloxacin-resistant mutants of staphylococcus aureus. *Antimicrobial Agents and Chemotherapy*, 39(7):1554–1558, July 1995. PMID: 7492103 PMCID: 162780.
20. Lisa Friedman, Jeff D. Alder, and Jared A. Silverman. Genetic changes that correlate with reduced susceptibility to daptomycin in staphylococcus aureus. *Antimicrobial Agents and Chemotherapy*, 50(6):2137–2145, June 2006. PMID: 16723576 PMCID: 1479123.
21. Laura Garca-lvarez, Matthew TG Holden, Heather Lindsay, Cerian R Webb, Derek FJ Brown, Martin D Curran, Enid Walpole, Karen Brooks, Derek J Pickard, Christopher Teale, Julian Parkhill, Stephen D Bentley, Giles F Edwards, E Kirsty Girvan, Angela M Kearns, Bruno Pichon, Robert LR Hill, Anders Rhod Larsen, Robert L Skov, Sharon J Peacock, Duncan J Maskell, and Mark A Holmes. Meticillin-resistant staphylococcus aureus with a novel mecA homologue in human and bovine populations in the UK and denmark: a descriptive study. *The Lancet Infectious Diseases*, 11(8):595–603, August 2011.
22. Steven R Gill, Derrick E Fouts, Gordon L Archer, Emmanuel F Mongodin, Robert T DeBoy, Jacques Ravel, Ian T Paulsen, James F Kolonay, Lauren Brinkac, Mauren Beanan, Robert J Dodson, Sean C Daugherty, Ramana Madupu, Samuel V Angiuoli, A Scott Durkin, Daniel H Haft, Jessica Vamathevan, Hoda Khouri, Terry Utterback, Chris Lee, George Dimitrov, Lingxia Jiang, Haiying Qin, Jan Weidman, Kevin Tran, Kathy Kang, Ioana R Hance, Karen E Nelson, and Claire M Fraser. Insights on evolution of virulence and resistance from the complete genome analysis of an early Methicillin-Resistant staphylococcus aureus strain and a Biofilm-Producing Methicillin-Resistant staphylococcus epidermidis strain. *Journal of Bacteriology*, 187(7):2426–2438, April 2005.
23. H. Hanaki, K. Kuwahara-Arai, S. Boyle-Vavra, R S Daum, H. Labischinski, and K. Hiramatsu. Activated cell-wall synthesis is associated with vancomycin resistance in methicillin-resistant staphylococcus aureus clinical strains mu3 and mu50. *Journal of Antimicrobial Chemotherapy*, 42(2):199–209, August 1998.
24. Lisa Herron-Olson, J. Ross Fitzgerald, James M. Musser, and Vivek Kapur. Molecular correlates of host specialization in staphylococcus aureus. *PLoS ONE*, 2(10):e1120, October 2007.
25. Ellie Hershberger, Jeffrey R Aeschlimann, Tabitha Moldovan, and Michael J Rybak. Evaluation of bactericidal activities of LY333328, vancomycin, teicoplanin, Ampicillin-Sulbactam, trovafloxacin, and RP59500 alone or in combination with rifampin or gentamicin against different strains of Vancomycin-Intermediate staphylococcus aureus by Time-Kill curve methods. *Antimicrobial Agents and Chemotherapy*, 43(3):717–721, March 1999.
26. Sarah K. Highlander, Kristina G. Hulten, Xiang Qin, Huaiyang Jiang, Shailaja Yerrapragada, Edward O. Mason, Yue Shang, Tiffany M. Williams, Rgine M. Fortunov, Yamei Liu, Okezie Igboeli, Joseph Petrosino, Madhan Tirumalai, Akif Uzman, George E. Fox, Ana Maria M. Cardenas, Donna M. Muzny, Lisa Hemphill, Yan Ding, Shannon Dugan, Peter R. Blyth, Christian J. Buhay, Huyen H. Dinh, Alicia C. Hawes, Michael Holder, Christie L. Kovar, Sandra L. Lee, Wen Liu, Lynne V. Nazareth, Qiaoyan Wang, Jianling Zhou, Sheldon L. Kaplan, and George M. Weinstock. Subtle genetic changes enhance virulence of methicillin resistant and sensitive \textitStaphylococcus aureus. *BMC microbiology*, 7(1):99+, November 2007.
27. Matthew T. G Holden, Edward J Feil, Jodi A Lindsay, Sharon J Peacock, Nicholas P. J Day, Mark C Enright, Tim J Foster, Catrin E Moore, Laurence Hurst, Rebecca Atkin, Andrew Barron, Nathalie Bason, Stephen D Bentley, Carol Chillingworth, Tracey Chillingworth, Carol Churcher, Louise Clark, Craig Corton, Ann Cronin, Jon Doggett, Linda Dowd, Theresa Feltwell, Zahra Hance, Barbara Harris, Heidi Hauser, Simon Holroyd, Kay Jagels, Keith D James, Nicola Lennard, Alexandra Line, Rebecca Mayes, Sharon Moule, Karen Mungall, Douglas Ormond, Michael A Quail, Ester Rabbinowitsch, Kim Rutherford, Mandy Sanders, Sarah Sharp, Mark Simmonds, Kim Stevens, Sally Whitehead, Bart G Barrell, Brian G Spratt, and Julian Parkhill. Complete genomes of two clinical staphylococcus aureus strains: Evidence for the rapid evolution of virulence and drug resistance. *Proceedings of the* [illegible]
28. Matthew T. G Holden, Jodi A Lindsay, Craig Corton, Michael A Quail, Joshua D Cockfield, Smriti Pathak, Rahul Batra, Julian Parkhill, Stephen D Bentley, and Jonathan D Edgeworth. Genome sequence of a recently emerged, highly transmissible, Multi-Antibiotic- and Antiseptic-Resistant variant of Methicillin-Resistant staphylococcus aureus, sequence type 239 (TW). *Journal of Bacteriology*, 192(3):888–892, February 2010.
29. Deborah C. Holt, Matthew T. G. Holden, Steven Y. C. Tong, Santiago CastilloRamirez, Louise Clarke, Michael A. Quail, Bart J. Currie, Julian Parkhill, Stephen D. Bentley, Edward J. Feil, and Philip M. Giffard. A very EarlyBranching \textitStaphylococcus aureus lineage lacking the carotenoid pigment staphyloxanthin. *Genome Biology and Evolution*, 3:881895, January 2011.
30. Benjamin P Howden, Paul D R Johnson, Peter B Ward, Timothy P Stinear, and John K Davies. Isolates with Low-Level vancomycin resistance associated with persistent Methicillin-Resistant staphylococcus aureus bacteremia. *Antimicrobial Agents and Chemotherapy*, 50(9):3039–3047, September 2006.
31. Benjamin P Howden, Torsten Seemann, Paul F Harrison, Chris R McEvoy, Jo-Ann L Stanton, Christy J Rand, Chris W Mason, Slade O Jensen, Neville Firth, John K Davies, Paul D R Johnson, and Timothy P Stinear. Complete genome sequence of staphylococcus aureus strain JKD6008, an ST239 clone of Methicillin-Resistant staphylococcus aureus with Intermediate-Level vancomycin resistance. *Journal of Bacteriology*, 192(21):5848–5849, November 2010.
32. Benjamin P. Howden, Timothy P. Stinear, David L. Allen, Paul D. R. Johnson, Peter B. Ward, and John K. Davies. Genomic analysis reveals a point mutation in the TwoComponent sensor gene graS that leads to intermediate vancomycin resistance in clinical \textitStaphylococcus aureus. *Antimicrobial Agents and Chemotherapy*, 52(10):37553762, 2008.
33. Adam D Kennedy, Stephen F Porcella, Craig Martens, Adeline R Whitney, Kevin R Braughton, Liang Chen, Carly T Craig, Fred C Tenover, Barry N Kreiswirth, James M Musser, and Frank R DeLeo. Complete nucleotide sequence analysis of plasmids in strains of staphylococcus aureus clone USA300 reveals a high level of identity among isolates with closely related core genome sequences. *Journal of Clinical Microbiology*, 48(12):4504–4511, December 2010.
34. M Kuroda, K Kuwahara-Arai, and K Hiramatsu. Identification of the up- and down-regulated genes in vancomycin-resistant staphylococcus aureus strains mu3 and mu50 by cDNA differential hybridization method. *Biochemical and Biophysical Research Communications*, 269(2):485–490, March 2000. PMID: 10708580.
35. M. Kuroda, T. Ohta, I. Uchiyama, T. Baba, H. Yuzawa, and Others. Whole genome sequencing of meticillinresistant \textitStaphylococcus aureus. *Lancet*, 357(9264):12251240, 2001.
36. Makoto Kuroda, Hiroko Kuroda, Taku Oshima, Fumihiko Takeuchi, Hirotada Mori, and Keiichi Hiramatsu. Two-component system VraSR positively modulates the regulation of cell-wall biosynthesis pathway in staphylococcus aureus. *Molecular Microbiology*, 49(3):807–821, August 2003. PMID: 12864861.
37. Kerry L LaPlante, Steven N Leonard, David R Andes, William A Craig, and Michael J Rybak. Activities of clindamycin, daptomycin, doxycycline, linezolid, Trimethoprim-Sulfamethoxazole, and vancomycin against Community-Associated Methicillin-Resistant staphylococcus aureus with inducible clindamycin resistance in murine thigh infection and in vitro pharmacodynamic models. *Antimicrobial Agents and Chemotherapy*, 52(6):2156–2162, June 2008.
38. Mark Joon-Sung Lee. *Phenotypic and genetic characterization of borderline oxacillin-resistant Staphylococcus aureus*. University of Toronto, 1999.
39. Yang Li, Boyang Cao, Yongbi Zhang, Jiali Zhou, Bin Yang, and Lei Wang. Complete genome sequence of \textitStaphylococcus aureus t0131, an ST239MRSASCCmec type III clone isolated in china. *Journal of Bacteriology*, 193(13):34113412, 2011.
40. YingChi Lin, Michele J. Anderson, Petra L. Kohler, Kristi L. Strandberg, Michael E. Olson, Alexander R. Horswill, Patrick M. Schlievert, and Marnie L. Peterson. Proinflammatory exoprotein characterization of toxic shock syndrome \textitStaphylococcus aureus. *Biochemistry*, 50(33):71577167, 2011.
41. M. Lindqvist, B. Isaksson, C. Grub, T. Jonassen, and A. Hllgren. Detection and characterisation of SCC<i>mec</i> remnants in multiresistant methicillin-susceptible <i>Staphylococcus aureus</i> causing a clonal outbreak in a swedish county. *European Journal of Clinical Microbiology & Infectious Diseases*, 31(2):141–147, February 2012.
42. Fionnuala M. McAleese and Timothy J. Foster. Analysis of mutations in the \textitStaphylococcus aureus \textitclfB promoter leading to increased expression. *Microbiology*, 149(1):99109, 2003.
43. Michael Meehl, Silvia Herbert, Friedrich Gotz, and Ambrose Cheung. Interaction of the \textitgraRS TwoComponent system with the \textitvraFG ABCTransporter to support VancomycinIntermediate resistance in \textitStaphylococcus aureus. *Antimicrobial Agents and Chemotherapy*, 51(8):27692689, 2007.
44. Robert E. Molestina. BEI resources: a biological resource center for parasitologists. *Trends in Parasitology*, October 2010.
45. M. M. Mwangi, S. W. Wu, Y. Zhou, K. Sieradzki, H. de Lencastre, P. Richardson, D. Bruce, E. Rubin, E. Myers, E. D. Siggia, and A. Tomasz. Tracking the in vivo evolution of multidrug resistance in \textitStaphylococcus aureus by wholegenome sequencing. *Proceedings of the National Academy of Sciences*, 104(22):94519456, May 2007.
46. Ulrich Nbel, Janina Dordel, Kevin Kurt, Birgit Strommenger, Henrik Westh, Sanjay K. Shukla, Helena emlikov, Raphal Leblois, Thierry Wirth, Thibaut Jombart, Franois Balloux, and Wolfgang Witte. A timescale for evolution, population expansion, and spatial spread of an emerging clone of Methicillin-Resistant staphylococcus aureus. *PLoS Pathog*, 6(4):e1000855, April 2010.
47. Hui-min Neoh, Longzhu Cui, Harumi Yuzawa, Fumihiko Takeuchi, Miki Matsuo, and Keiichi Hiramatsu. Mutated response regulator graR is responsible for phenotypic conversion of staphylococcus aureus from heterogeneous Vancomycin-Intermediate resistance to Vancomycin-Intermediate resistance. *Antimicrobial Agents and Chemotherapy*, 52(1):45–53, January 2008. PMID: 17954695 PMCID: 2223914.
48. Federico G Nicola, Linda K McDougal, James W Biddle, and Fred C Tenover. Characterization of Erythromycin-Resistant isolates of staphylococcus aureus recovered in the united states from 1958 through 1969. *Antimicrobial Agents and Chemotherapy*, 42(11):3024–3027, November 1998.
49. Brunello Oliva, Alexander John ONeill, Keith Miller, William Stubbings, and Ian Chopra. Anti-staphylococcal activity and mode of action of clofazimine. *Journal of Antimicrobial Chemotherapy*, 53(3):435–440, March 2004.
50. A. J. O'Neill, T. Huovinen, C. W. G. Fishwick, and I. Chopra. Molecular genetic and structural modeling studies of \textitStaphylococcus aureus RNA polymerase and the fitness of rifampin resistance genotypes in relation to clinical prevalence. *Antimicrobial Agents and Chemotherapy*, 50(1):298309, 2006.
51. Alexander J ONeill and Ian Chopra. Insertional inactivation of mutS in staphylococcus aureus reveals potential for elevated mutation frequencies, although the prevalence of mutators in clinical isolates is low. *Journal of Antimicrobial Chemotherapy*, 50(2):161–169, August 2002.
52. Anton Y. Peleg, Spiros Miyakis, Doyle V. Ward, Ashlee M. Earl, Aileen Rubio, David R. Cameron, Satish Pillai, Robert C. Moellering, and George M. Eliopoulos. Whole genome characterization of the mechanisms of daptomycin resistance in clinical and laboratory derived isolates of staphylococcus aureus. *PLoS ONE*, 7(1):e28316, January 2012.
53. Milla Pietiainen, Patrice Francois, Hanne-Leena Hyyrylainen, Mamella Tangomo, Vera Sass, Hans-Georg Sahl, Jacques Schrenzel, and Vesa P Kontinen. Transcriptome analysis of the responses of staphylococcus aureus to antimicrobial peptides and characterization of the roles of vraDE and vraSR in antimicrobial resistance. *BMC Genomics*, 10:429, September 2009. PMID: 19751498 PMCID: 2748101.
54. Satish K Pillai, Christine Wennersten, Lata Venkataraman, George M Eliopoulos, Robert C Moellering, and Adolf W Karchmer. Development of reduced vancomycin susceptibility in Methicillin-Susceptible staphylococcus aureus. *Clinical Infectious Diseases*, 49(8):11691174, October 2009.
55. Michael J Pucci and Thomas J Dougherty. Direct quantitation of the numbers of individual Penicillin-Binding proteins per cell in staphylococcus aureus. *Journal of Bacteriology*, 184(2):588–591, January 2002.
56. D Ashley Robinson, Angela M Kearns, Anne Holmes, Donald Morrison, Hajo Grundmann, Giles Edwards, Frances G O'Brien, Fred C Tenover, Linda K McDougal, Alastair B Monk, and Mark C Enright. Re-emergence of early pandemic staphylococcus aureus as a community-acquired meticillin-resistant clone. *Lancet*, 365(9466):1256–1258, April 2005. PMID: 15811459.
57. Jean-Marc Rolain, Patrice Franois, David Hernandez, Fadi Bittar, Herv Richet, Ghislain Fournous, Yves Mattenberger, Emmanuelle Bosdure, Nathalie Stremler, Jean-Christophe Dubus, Jacques Sarles, Martine Reynaud-Gaubert, Stephanie Boniface, Jacques Schrenzel, and Didier Raoult. Genomic analysis of an emerging multiresistant staphylococcus aureus strain rapidly spreading in cystic fibrosis patients revealed the presence of an antibiotic inducible bacteriophage. *Biology Direct*, 4:1, 2009. PMID: 19144117.
58. George Sakoulas, Jeff Alder, Claudio Thauvin-Eliopoulos, Robert C. Moellering, and George M. Eliopoulos. Induction of daptomycin heterogeneous susceptibility in staphylococcus aureus by exposure to vancomycin. *Antimicrobial Agents and Chemotherapy*, 50(4):1581–1585, April 2006. PMID: 16569891 PMCID: 1426932.
59. Franziska Schaaff, Andrea Reipert, and Gabriele Bierbaum. An elevated mutation frequency favors development of vancomycin resistance in staphylococcus aureus. *Antimicrobial Agents and Chemotherapy*, 46(11):3540–3548, November 2002. PMID: 12384362 PMCID: 128741.
60. Maarten J Schijffelen, CH Edwin Boel, Jos AG van Strijp, and Ad C Fluit. Whole genome analysis of a livestock-associated methicillin-resistant staphylococcus aureus ST398 isolate from a case of human endocarditis. *BMC Genomics*, 11(1):376, June 2010.
61. K. Sieradzki, T. Leski, J. Dick, L. Borio, and A. Tomasz. Evolution of a Vancomycin-Intermediate staphylococcus aureus strain in vivo: Multiple changes in the antibiotic resistance phenotypes of a single lineage of Methicillin-Resistant s. aureus under the impact of antibiotics administered for chemotherapy. *Journal of Clinical Microbiology*, 41(4):1687–1693, April 2003. PMID: 12682161 PMCID: 153915.
62. Tara C. Smith, Michael J. Male, Abby L. Harper, Jennifer S. Kroeger, Gregory P. Tinkler, Erin D. Moritz, Ana W. Capuano, Loreen A. Herwaldt, and Daniel J. Diekema. Methicillin-Resistant staphylococcus aureus (MRSA) strain ST398 is present in midwestern U.S. swine and swine workers. *PLoS ONE*, 4(1):e4258, January 2009.
63. E. E. Snyder, N. Kampanya, J. Lu, E. K. Nordberg, H. R. Karur, M. Shukla, J. Soneja, Y. Tian, T. Xue, H. Yoo, F. Zhang, C. Dharmanolla, N. V. Dongre, J. J. Gillespie, J. Hamelius, M. Hance, K. I. Huntington, D. Jukneliene, J. Koziski, L. Mackasmiel, S. P. Mane, V. Nguyen, A. Purkayastha, J. Shallom, G. Yu, Y. Guo, J. Gabbard, D. Hix, A. F. Azad, S. C. Baker, S. M. Boyle, Y. Khudyakov, X. J. Meng, C. Rupprecht, J. Vinje, O. R. Crasta, M. J. Czar, A. Dickerman, J. D. Eckart, R. Kenyon, R. Will, J. C. Setubal, and B. W. S. Sobral. PATRIC: the VBI PathoSystems resource integration center. *Nucleic Acids Research*, 35(Database issue):D401–D406, January 2007. PMID: 17142235 PMCID: 1669763.
64. Marc Stegger, Lance B Price, Anders R Larsen, John D Gillece, Andrew E Waters, Robert Skov, and Paal S Andersen. Genome sequence of staphylococcus aureus strain 11819-97, an ST80-IV european Community-Acquired Methicillin-Resistant isolate. *Journal of Bacteriology*, 194(6):1625–1626, March 2012.
65. M J Storrs, P Courvalin, and T J Foster. Genetic analysis of gentamicin resistance in methicillin- and gentamicin-resistant strains of staphylococcus aureus isolated in dublin hospitals. *Antimicrobial Agents and Chemotherapy*, 32(8):1174–1181, August 1988. PMID: 2847646 PMCID: 172372.
66. Masahiro Suzuki, Kazuhiro Yamada, Miki Nagao, Etsuko Aoki, Masakado Matsumoto, Tatsuya Hirayama, Hiroaki Yamamoto, Reiji Hiramatsu, Satoshi Ichiyama, and Yoshitsugu Iinuma. Antimicrobial ointments and methicillin-resistant staphylococcus aureus USA300. *Emerging Infectious Diseases*, 17(10):1917–1920, October 2011. PMID: 22000371.
67. Fred C Tenover, Michael V Lancaster, Bertha C Hill, Christine D Steward, Sheila A Stocker, Gary A Hancock, Caroline M OHara, Nancye C Clark, and Keiichi Hiramatsu. Characterization of staphylococci with reduced susceptibilities to vancomycin and other glycopeptides. *Journal of Clinical Microbiology*, 36(4):1020–1027, April 1998.
68. Anne W WYKE, J. Barrie WARD, and Michael V HAYES. Synthesis of peptidoglycan in vivo in MethicillinResistant staphylococcus aureus. *European Journal of Biochemistry*, 127(3):553–558, October 1982.
69. Huping Xue, Hong Lu, and Xin Zhao. Sequence diversities of Serine-Aspartate repeat genes among staphylococcus aureus isolates from different hosts presumably by horizontal gene transfer. *PLoS ONE*, 6(5):e20332, May 2011.
70. H. Yamada, S. Kurose-Hamada, Y. Fukuda, J. Mitsuyama, M. Takahata, S. Minami, Y. Watanabe, and H. Narita. Quinolone susceptibility of norA-Disrupted staphylococcus aureus. *Antimicrobial Agents and Chemotherapy*, 41(10):2308–2309, October 1997.
71. Tatsuo Yamamoto, Tomomi Takano, Wataru Higuchi, Yasuhisa Iwao, Olga Singur, Ivan Reva, Yuta Otsuka, Toru Nakayashiki, Hirotada Mori, Galina Reva, Vladimir Kuznetsov, and Vladimir Potapov. Comparative genomics and drug resistance of a geographic variant of ST239 Methicillin-Resistant staphylococcus aureus emerged in russia. *PLoS ONE*, 7(1):e29187, January 2012.
